# Supplementary material for: Identifying Patients at Risk of Acute Kidney Injury among Patients Receiving Immune Checkpoint Inhibitors: A Machine Learning Approach
Source: Diagnostics (Basel). 2022 Dec 14;12(12):3157. doi: 10.3390/diagnostics12123157 (PMC9776989; doi:10.3390/diagnostics12123157)
Supplement: Supplementary file 1 [file diagnostics-12-03157-s001.zip › diagnostics-2038417-supplementary.pdf]

**Table S1.** The performance comparisons of different models based on all variables

| Model | AUROC | P-Value | VS |
|---|---|---|---|
| Support Vector Machine (SVM) with radial kernel | 0.8093 | 0.6194 | Support Vector Machine (SVM) with sigmoid kernel |
| | | 0.9154 | Support Vector Machine (SVM) with polynomial kernel |
| | | 0.6437 | Decision Tree (DT) |
| | | 0.6761 | Random Forest (RF) |
| | | 0.1898 | logistic Regression (LR) |
| | | 0.866 | Neural Networks (NNs) |
| | | 0.8664 | Adaptive Boosting (Adaboost) |
| | | 0.3774 | Extreme Gradient Boosting (XGboost) |
| | | 0.4691 | Naïve Bayes (NBs) |
| Support Vector Machine (SVM) with sigmoid kernel | 0.7787 | 0.4426 | Support Vector Machine (SVM) with polynomial kernel |
| | | 0.967 | Decision Tree (DT) |
| | | 0.4733 | Random Forest (RF) |
| | | 0.2728 | logistic Regression (LR) |
| | | 0.4856 | Neural Networks (NNs) |
| | | 0.7685 | Adaptive Boosting (Adaboost) |
| | | 0.8649 | Extreme Gradient Boosting (XGboost) |
| | | 0.9099 | Naïve Bayes (NBs) |
| Support Vector Machine (SVM) with polynomial kernel | 0.813 | 0.5606 | Decision Tree (DT) |
| | | 0.7991 | Random Forest (RF) |
| | | 0.1058 | logistic Regression (LR) |
| | | 0.9233 | Neural Networks (NNs) |
| | | 0.8266 | Adaptive Boosting (Adaboost) |
| | | 0.3146 | Extreme Gradient Boosting (XGboost) |
| | | 0.5017 | Naïve Bayes (NBs) |
| Decision Tree (DT) | 0.7764 | 0.4493 | Random Forest (RF) |
| | | 0.4246 | logistic Regression (LR) |
| | | 0.559 | Neural Networks (NNs) |
| | | 0.732 | Adaptive Boosting (Adaboost) |
| | | 0.874 | Extreme Gradient Boosting (XGboost) |
| | | 0.9009 | Naïve Bayes (NBs) |

| | | | |
|---|---|---|---|
| Random Forest (RF) | 0.8241 | 0.1378 | logistic Regression (LR) |
| | | 0.8816 | Neural Networks (NNs) |
| | | 0.5983 | Adaptive Boosting (Adaboost) |
| | | 0.1352 | Extreme Gradient Boosting (XGboost) |
| | | 0.3358 | Naïve Bayes (NBs) |
| logistic Regression (LR) | 0.7204 | 0.1072 | Neural Networks (NNs) |
| | | 0.2886 | Adaptive Boosting (Adaboost) |
| | | 0.4627 | Extreme Gradient Boosting (XGboost) |
| | | 0.2967 | Naïve Bayes (NBs) |
| Neural Networks (NNs) | 0.8167 | 0.7947 | Adaptive Boosting (Adaboost) |
| | | 0.3802 | Extreme Gradient Boosting (XGboost) |
| | | 0.507 | Naïve Bayes (NBs) |
| Adaptive Boosting (Adaboost) | 0.8 | 0.489 | Extreme Gradient Boosting (XGboost) |
| | | 0.8244 | Naïve Bayes (NBs) |
| Extreme Gradient Boosting (XGboost) | 0.7685 | 0.7609 | Naïve Bayes (NBs) |

**Table S2.** The performance comparisons of different models based on important variables

| Model | AUROC | P-Value | VS |
|---|---|---|---|
| Support Vector Machine (SVM) with radial kernel | 0.8315 | 0.9345 | Support Vector Machine (SVM) with sigmoid kernel |
| | | 0.7414 | Support Vector Machine (SVM) with polynomial kernel |
| | | 0.643 | Decision Tree (DT) |
| | | 0.9654 | Random Forest (RF) |
| | | 0.1799 | logistic Regression (LR) |
| | | 0.2407 | Neural Networks (NNs) |
| | | 0.4175 | Adaptive Boosting (Adaboost) |
| | | 0.1077 | Extreme Gradient Boosting (XGboost) |
| | | 0.301 | Naïve Bayes (NBs) |
| Support Vector Machine (SVM) with sigmoid kernel | 0.8217 | 0.6636 | Support Vector Machine (SVM) with polynomial kernel |
| | | 0.6272 | Decision Tree (DT) |
| | | 0.9562 | Random Forest (RF) |
| | | 0.1552 | logistic Regression (LR) |
| | | 0.2208 | Neural Networks (NNs) |
| | | 0.508 | Adaptive Boosting (Adaboost) |
| | | 0.2045 | Extreme Gradient Boosting (XGboost) |
| | | 0.5514 | Naïve Bayes (NBs) |
| Support Vector Machine (SVM) with polynomial kernel | 0.8213 | 0.7414 | Decision Tree (DT) |
| | | 0.7726 | Random Forest (RF) |
| | | 0.2144 | logistic Regression (LR) |
| | | 0.3257 | Neural Networks (NNs) |
| | | 0.6447 | Adaptive Boosting (Adaboost) |
| | | 0.312 | Extreme Gradient Boosting (XGboost) |
| | | 0.6537 | Naïve Bayes (NBs) |
| Decision Tree (DT) | 0.8056 | 0.6729 | Random Forest (RF) |
| | | 0.5016 | logistic Regression (LR) |
| | | 0.6041 | Neural Networks (NNs) |
| | | 0.9439 | Adaptive Boosting (Adaboost) |
| | | 0.6144 | Extreme Gradient Boosting (XGboost) |
| | | 0.9789 | Naïve Bayes (NBs) |

| | | | |
|---|---|---|---|
| Random Forest (RF) | 0.8306 | 0.1572 | logistic Regression (LR) |
| | | 0.2465 | Neural Networks (NNs) |
| | | 0.3167 | Adaptive Boosting (Adaboost) |
| | | 0.09311 | Extreme Gradient Boosting (XGboost) |
| | | 0.3644 | Naïve Bayes (NBs) |
| logistic Regression (LR) | 0.7528 | 0.7985 | Neural Networks (NNs) |
| | | 0.435 | Adaptive Boosting (Adaboost) |
| | | 0.7757 | Extreme Gradient Boosting (XGboost) |
| | | 0.2635 | Naïve Bayes (NBs) |
| Neural Networks (NNs) | 0.769 | 0.6027 | Adaptive Boosting (Adaboost) |
| | | 0.9717 | Extreme Gradient Boosting (XGboost) |
| | | 0.5368 | Naïve Bayes (NBs) |
| Adaptive Boosting (Adaboost) | 0.8099 | 0.4213 | Extreme Gradient Boosting (XGboost) |
| | | 0.9523 | Naïve Bayes (NBs) |
| Extreme Gradient Boosting (XGboost) | 0.7713 | 0.4968 | Naïve Bayes (NBs) |
